# Short-term clinical outcomes of Kyocera Modular Limb Salvage System designed cementless stems for the endoprosthetic reconstruction of lower extremities: a Japanese Musculoskeletal Oncology Group multi-institutional study

**DOI:** 10.1186/s12885-022-09873-x

**Published:** 2022-07-16

**Authors:** Satoshi Tsukushi, Yoshihiro Nishida, Takeshi Hirose, Eiji Nakata, Rumi Nakagawa, Tomoki Nakamura, Jungo Imanishi, Akihito Nagano, Hironari Tamiya, Takafumi Ueda, and Japanese Musculoskeletal Oncology Group (JMOG), Satoshi Tsukushi, Yoshihiro Nishida, Kunihiro Ikuta, Akira Kawai, Takeshi Hirose, Toshiyuki Kunisada, Eiji Nakata, Robert Nakayama, Rumi Nakagawa, Tomoki Nakamura, Tomoaki Torigoe, Jungo Imanishi, Akihito Nagano, Satoshi Takenaka, Hironari Tamiya, Takafumi Ueda, Shigeki Kakunaga, Hirotaka Kawano, Toshiharu Shirai, Ryu Terauchi, Hidetatsu Outani, Shunji Nishimura, Kanya Honoki

**Affiliations:** 1 Department of Orthopaedic Surgery, Aichi Cancer Center Hospital, 1-1 Kanokoden, Chikusa-ku Nagoya, 464-8681 Japan; 2 Department of Rehabilitation Medicine, Nagoya University Hospital, Nagoya, Japan; 3 Department of Musculoskeletal Oncology, National Cancer Center Hospital, Tokyo, Japan; 4 Department of Orthopaedic Surgery, Okayama University Graduate School of Medicine, Dentistry, and Pharmaceutical Sciences, Okayama, Japan; 5 Department of Orthopaedic Surgery, Keio University School of Medicine, Tokyo, Japan; 6 Department of Orthopedic Surgery, Mie University Graduate School of Medicine, Tsu, Japan; 7 Department of Orthopaedic Oncology and Surgery, Saitama Medical University International Medical Center, Hidaka, Japan; 8 Department of Orthopaedic Surgery, Gifu University School of Medicine, Gifu, Japan; 9 Department of Orthopedic Surgery, Osaka International Cancer Institute, Osaka, Japan; 10 Department of Orthopaedic Surgery, National Hospital Organization Osaka National Hospital, Osaka, Japan

**Keywords:** Endoprosthesis, Cementless stem, Complication, Stress shielding

## Abstract

**Background:**

The high rate of aseptic loosening of cemented stems has led to their frequent use in endoprosthetic reconstruction. However, problems, such as stem breakage and stress shielding at the insertion site, remain. The Japanese Musculoskeletal Oncology Group (JMOG) has developed Kyocera Modular Limb Salvage System (KMLS) cementless stems with a unique tapered press-fit and short fixation design. This study aimed to clarify the short-term postoperative outcomes of this prosthesis and validate the stem design.

**Methods:**

One hundred cases of KMLS cementless stems (51 male patients; median age, 49 years; mean follow-up period, 35 months), with a minimum follow-up of 2 years, for the proximal femur (PF), distal femur (DF), and proximal tibia were prospectively registered for use. Prosthesis survival, complication rates, postoperative functional, and radiographical evaluation were analyzed. Complications or failures after insertion of the KMLS endoprostheses were classified into five types and functional results were analyzed according to the MSTS scoring system at postoperative 1 year. The diaphyseal interface and anchorage were graded by the ISOLS system at postoperative 2 years.

**Results:**

The overall prosthesis survival rates at 2 and 4 years were 88.2 and 79.6%, respectively. The prosthesis-specific survival rate excluding infection and tumor recurrence was 90.2 and 87.9%, respectively. Younger age (*p* = 0.045) and primary tumor (*p* = 0.057) were associated with poor prognosis of prosthesis-specific survival excluding infection and tumor recurrence. Complications were observed in 31 patients, 13 patients underwent revision surgery. The mean MSTS functional score at 1 year postoperatively was 68%. Early implant loosening was significantly more common in the DF (*p* = 0.006) and PF/DF straight stem (*p* = 0.038). The ISOLS radiographic evaluation at 2 years after surgery revealed good bone remodeling and anchorage in most cases (bone remodeling: 90% / excellent and good, anchorage: 97% / excellent and good).

**Conclusions:**

Tumor endoprosthesis long-term fixation to the diaphysis of the lower extremity remains challenging. The KMLS cementless stem with a unique tapered press fit design showed good short-term results in maintaining bone stock. To prevent early loosening, a curved stem should be used in PF and DF, but long-term follow-up is necessary.

## Background

Due to advances in multidisciplinary approaches for malignant bone tumors, such as Ewing’s sarcoma and osteosarcoma, and the development of various endoprostheses, limb-salvage surgery has become the gold standard [1, 2]. The prognosis for patients with musculoskeletal sarcomas has improved significantly over the past three decades. Consequently, the durability and longevity of these endoprosthetic implants are of great importance [3–6].

Long-term fixation of the tumor endoprosthesis to the diaphysis of long bones is very challenging, and the optimal method remains controversial. These endoprostheses endure very high rotational stresses at the bone-prosthesis interface due to various factors (young age, high levels of physical activity, loss of long segments, loss of static ligamentous stabilizers, and extensive muscle resection). Historically, cemented prostheses were used due to their immediate stability, which allows for early weight-bearing [7–13]. However, further studies have shown high rates of aseptic loosening in patients with long-term follow-up [4, 10, 13, 14]. The reported prosthetic survivorship was 88% at 2 years, 82% at 5 years, and 59% at 10 years [4]. Therefore, orthopedic oncologists frequently use cementless stems to improve long-term implant survival [15–17].

The early cementless stems incorporated a side plate and screw components. As it is associated with very high rates of intramedullary fixation, aseptic loosening is rare but various failures such as stem breakage, screw loosening, and stress shielding are often experienced [16–19]. Various forms of press-fit cementless stem with or without flutes have been developed [20–22]. Most press-fit cementless stems have long extensively porous-coated intramedullary stems allowing bony growth over the whole length of the stem. However, intramedullary fixation is inherently unphysiological and results in reduced loading of the surrounding cortical bone. This results in stress shielding of the surrounding bone and an increased risk of aseptic loosening. However, the optimal design to reduce stress shielding is controversial.

Tumor endoprostheses that are used worldwide are generally designed for Caucasian body types and are frequently too large or too heavy for Asian-pacific patients. The Japanese Musculoskeletal Oncology Group (JMOG) has been involved in modifying small and light modular prothesis (Physio Hinge Total Knee System Type III/PHK III,) that requires bone cement to fix the femoral stem [19, 23].. In 2002, the JMOG developed a new cementless stem in addition to the PHK III series and introduced the prosthesis as the Kyocera Modular Limb Salvage (KMLS) system. Currently, the KMLS system is used as a reconstructive prosthesis for the proximal femur, distal femur, and proximal tibia, with a choice of cemented or cementless stems depending on the patients. The early KMLS cementless stems incorporated a side plate and screw components, and their implant survival rates are generally consistent with those reported in the literature. However, various failures such as stem breakage, screw loosening, and stress shielding are often experienced [24]. Therefore, KMLS has developed a newly designed cementless stem with a unique tapered press-fit and short fixation design for the proximal femur (PF), distal femur (DF), and proximal tibia (PT) that has been in use since August 2014. From August 2014 to March 2018, 100 cases of KMLS newly designed cementless stems were prospectively registered for use at JMOG-affiliated institutions.

The purpose of this study was to clarify the short-term postoperative results of the cementless stem with a unique tapered press-fit design, confirm the validity of the stem design, and provide an index for future improvement.

## Methods

### Study design and setting

From August 2014 to March 2018, 100 cases of KMLS newly designed cementless stems for the PF, DF, and PT were prospectively registered for use at JMOG-affiliated 14 institutions. Following institutional review board approval, we retrospectively reviewed clinical outcomes of 100 newly designed cementless stems with a minimum follow-up of 2 years in March 2020.

The records of all patients were collected using a questionnaire administered to the members of the JMOG. The collected data included the demographic details, histological diagnosis, surgical stage, adjuvant therapy, size of the prosthetic components, complications, 1-year postoperative MSTS functional score, 2-year ISOLS radiographic evaluation, and oncological outcome at the final follow-up.

The primary endpoint of this study was to identify the prosthesis survival and the implant complication/failure rates. The secondary endpoint was to determine the radiological and functional outcomes.

### Demographics, description of study population

A total of 100 cases of KMLS newly designed cementless stems for the PF, DF, and PT fit the inclusion criteria (Table 1). There were 51 male and 49 female patients. The median age was 49 years. Follow-up was at a minimum of 2 years (mean, 35 months; range, 24–53 months). Four (4%) patients were lost to follow-up. Anatomical locations were as follows: PF (*n* = 49), DF (*n* = 39), and PT (*n* = 12). There were 64 primary tumors (57 bone tumors and 7 soft tissue sarcomas) and 36 metastatic bone tumors. The primary bone tumors included 35 osteosarcomas, eight chondrosarcomas, eight giant cell tumors of bone, five undifferentiated pleomorphic sarcomas, and one leiomyosarcoma. The primary soft tissue sarcomas included two undifferentiated pleomorphic sarcomas, two leiomyosarcoma, two synovial sarcomas, and one liposarcoma. Chemotherapy was administered in 57 patients and irradiation in three patients. Extra-articular resections were performed in seven patients of DF and a patient of PF. For the cementless stems of the PF and DF, straight and curved stems can be used selectively. In the PF, a straight stem was used in eight (16%) of 49 cases. In the DF, a straight stem was used in eight (21%) of 39 cases.

**Table 1:** Clinicopathological data on 100 cases of reconstruction with the newly designed cementless stem

Characteristic	All	Proximal	Distal	Proximal
		femur	femur	tibia
	(***N*** = 100)	(***N*** = 49)	(***N*** = 39)	(***N*** = 12)
Age				
median	49.0 yr	56.0 yr	30.0 yr	34.5 yr
≥50 yr	48	32	11	5
<50 yr	52	17	28	7
Gender				
male	51	19	25	7
female	49	30	14	5
Diagnosis				
primary	64	18	35	11
bone tumor	57	13	33	11
osteosarcoma	35	4	23	8
chondrosarcoma	8	5	3	0
giant cell tumor of bone	8	1	6	1
undifferentiated pleomorphic sarcoma	5	3	0	2
leiomyosarcoma	1	0	1	0
soft tissue tumor	7	5	2	0
undifferentiated pleomorphic sarcoma	2	2		
leiomyosarcoma	2	2		
synovial sarcoma	2	1	1	
liposarcoma	1		1	
metastatic bone tumor	36	31	4	1
BMI (body mass index)				
median	20.7	20.3	21.3	20.6
≥25	11	5	4	2
<25	89	44	35	10
With chemotherapy	57	26	24	7
Without chemotherapy	43	23	15	5
With radiation	3	2	1	0
Without radiation	97	47	38	12
diaphyseal/stem coefficient				
median	2.33	2.37	2.38	1.90
≥2.50	30	17	12	1
<2.50	70	32	27	11

### Prosthesis design

**Fig. 1:**
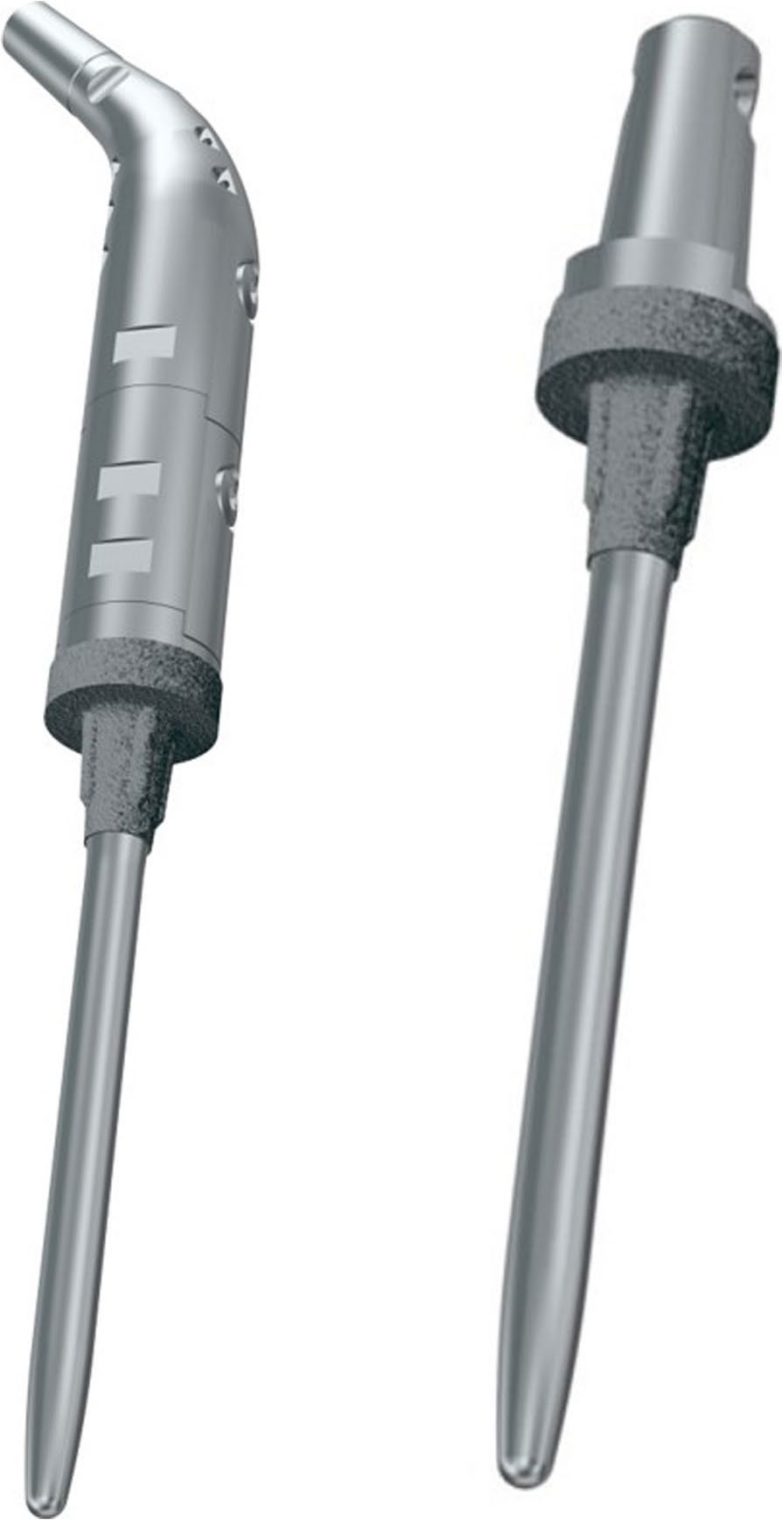
Detailed features of the KMLS newly designed cementless stems. The cementless stems of PF and DF have derotational flutes and straight and curved stems that can be used selectively. DF, Distal femur; KMLS, Kyocera Modular Limb Salvage System; PF, Proximal femur

The KMLS system is an original prosthesis of JMOG and is designed especially for patients with an Asian body type (Fig. 1). The metallic parts of the KMLS are made of lightweight and high-strength titanium alloy (Ti-6Al-4 V) with good biocompatibility and bio-stability. The base of the stem has a tapered press-fit design, and the interface is processed by porous proofing made of pure titanium to promote bone ingrowth. The titanium porous coating on the Ti-6AJ-4 V is performed with the inert gas-shielded arc spray at atmospheric pressure, which has been reported to show far fewer voids and cracks in the layers and interfacial areas between the layer and the substrate compared with low-pressure plasma spray coatings; this contributes to sound bone fixation at an early stage and to acceptable cementless joint fixation [25]. The cementless stems of the PF and DF have derotational flutes to provide adequate initial rotational stability, and straight and curved stems can be used selectively. The stem length is fixed at 125 mm for the femoral component and 110 mm for the tibial component. However, this prosthesis has not been approved for that use by the United States Food and Drug Administration.

### Variables, outcome measures, and data sources

**Fig. 2:**
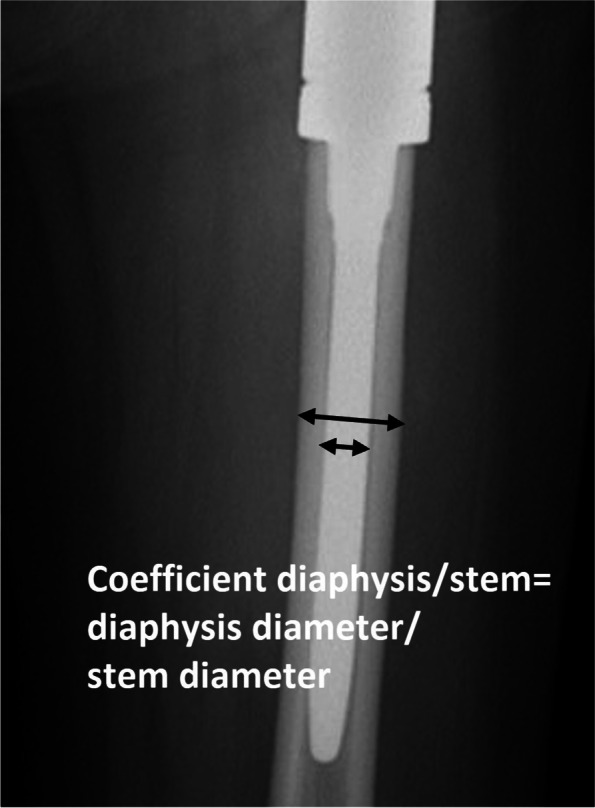
Coefficient diaphysis/stem. This coefficient was obtained through the division between the diaphysis diameter and the prosthetic-stem at the midpoint of the prosthetic-stem

We calculated the relative prosthetic-shaft diameter by dividing the diaphysis diameter by prosthetic diameter (diaphysis/stem coefficient) at the midpoint of the prosthetic stem (Fig. 2). It has been reported that a diaphyseal/stem coefficient of the cementless stem over 2.5 predicted lower prosthetic survival [26].

The diaphyseal interface and anchorage were graded using the ISOLS system [27] at postoperative 2 years; 65 of the 100 were available for ISOLS radiological evaluation (65%).

Functional results were analyzed according to the 30-point functional Musculoskeletal Tumor Society (MSTS) scoring system [28] at 1 year postoperatively; 72 of the 100 were available to assess the 1-year postoperative MSTS functional score (72%).

Complications or failures after insertion of the KMLS endoprostheses were classified according to Henderson *et al.* [29] in five different types: type 1 (mechanical failure due to soft tissue problems, such as debridement, peroneal nerve palsy, dislocation of joint [closed reduction]) and superficial infections), type 2 (aseptic loosening), type 3 (structural failures, such as periprosthetic fractures and hip dislocation requiring surgical treatment), type 4 (non-mechanical failures, such as deep infection), and type 5 (tumor progression).

### Statistical analysis

The statistical associations between the clinicopathological factors and complications were evaluated using Fisher’s exact test or the Chi-square test. The duration of survival was defined as the interval between the date of initial treatment for the primary tumor and the date of death. Patients who died from non-tumor-related causes were uncensored at the time of death in this study. The overall prosthesis survival rate was defined as the time from surgical reconstruction using the KMLS system to revision surgery due to any prosthetic failure including minor parts of the prosthesis, due to local recurrence, polyethylene bushing failure, breakage of the prosthesis, aseptic loosening, or infection. The prosthesis-specific survival rate was defined as the time from surgical reconstruction using the KMLS system to revision surgery due to implant failure excluding infection and tumor recurrence. The limb salvage rate was calculated as the time from surgical reconstruction using the KMLS system to amputation. Survival curves were constructed using the Kaplan–Meier method. The subgroups were compared using the log-rank test. The level of statistical significance was set at p < 0.05 and confidence intervals were reported at 95% (95% CI).

### Oncologic results

At the final follow-up, 59 patients were alive, and 41 patients had died of disease. The overall survival rates at 2 and 4 years were 88.2 and 79.6%, respectively. Twelve patients developed local recurrence. The limb salvage rate at 2 and 4 years was 98.9 and 96.9%, respectively. Two patients (one each with osteosarcoma and undifferentiated pleomorphic sarcoma of bone) underwent amputations for local recurrences.

## Results

### Prosthesis survival and prosthesis-specific survival

**Fig. 3:**
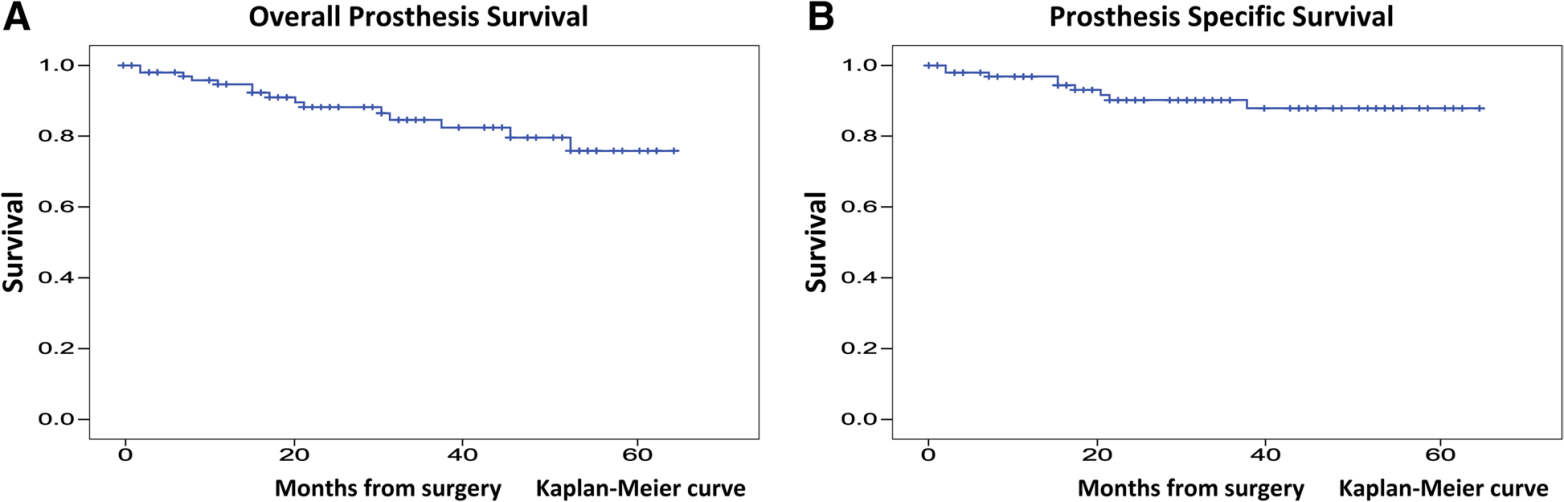
Survivorship of the KMLS newly designed cementless stem. **A**. Overall survivorship of the KMLS newly designed cementless stem. **B**. Prosthesis specific survivorship of the KMLS newly designed cementless stem. KMLS, Kyocera Modular Limb Salvage System

**Fig. 4:**
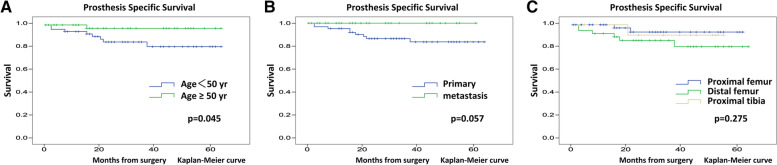
Kaplan–Meier survival curves showing prosthesis survival of the KMLS newly designed cementless stem in **A**. younger and older groups, **B**. primary and metastasis groups and **C**. in proximal femur, distal femur, and proximal tibia groups. KMLS, Kyocera Modular Limb Salvage System

The overall prosthesis survival rates at 2 and 4 years were 88.2 and 79.6%, respectively (Fig. 3A). Male sex (*p* = 0.063) and primary tumor (*p* = 0.08) were associated with poor prognosis of overall prosthesis survival (Table 2). The prosthesis-specific survival rate excluding infection and tumor recurrence at 2 and 4 years were 90.2 and 87.9%, respectively (Fig. 3B). Younger age (*p* = 0.045) and primary tumor (*p* = 0.057) were associated with poor prognosis of prosthesis-specific survival excluding infection and tumor recurrence (Table 3) (Fig. 4A-C).

**Table 2:** The relationship between overall prosthesis survival and patient characteristics

Variables		n	2-year survival (%)	***p*** value
Age	≥50 yr	48	94.6	*p* = 0.154
	<50 yr	52	83.4	
Gender	male	51	83.1	*p* = 0.063
	female	49	93.4	
Diagnosis	primary	64	85.1	*p* = 0.080
	metastasis	36	96.2	
Location	proximal femur	49	93.6	*p* = 0.279
	distal femur	39	81.5	
	proximal tibia	12	90.9	
BMI	≥25	11	79.5	*p* = 0.188
	<25	89	89.3	
Chemotherapy	yes	57	89.1	*p* = 0.783
	no	43	86.8	
Radiation	yes	3	100	*p* = 0.240
	no	97	87.8	
Diaphyseal/stem coefficient	≥2.50	30	84.3	*p* = 0.203
	<2.50	70	89.9

**Table 3:** The relationship between prosthesis specific-survival and patient characteristics

Variables		n	2-year survival (%)	***p*** value
Age	≥50 yr	48	97.1	*p* = 0.045*
	<50 yr	52	85.2	
Gender	male	51	85	*p* = 0.298
	female	49	95.5	
Diagnosis	primary	64	86.5	*p* = 0.057
	metastasis	36	100	
Location	proximal femur	49	93.6	*p* = 0.275
	distal femur	39	86.3	
	proximal tibia	12	80.9	
BMI	≥25	11	79.5	*p* = 0.246
	<25	89	96.3	
Chemotherapy	yes	57	90.9	*p* = 0.561
	no	43	89.3	
Radiation	yes	3	100	*p* = 0.622
	no	97	90	
Diaphyseal/stem coefficient	≥2.50	30	91.5	*p* = 0.630
	<2.50	70	89.9	

* a statistically significant *p* value

### Radiographic and functional evaluation

The ISOLS radiographic evaluation at 2 years after surgery revealed good bone remodeling and anchorage in most cases (72% excellent, 18% good, 8% fair, and 2% poor bone remodeling around the implant and 92% excellent, 5% good, and 2% fair for the anchorage itself). The mean MSTS functional score at 1 year postoperatively was 68%.

### Complication

In total, 31 failures/complications (31%) were observed in 100 KMLS newly designed cementless stems, and the anatomical location and failure classifications according to Henderson *et al.* are summarized in Table 4.

**Table 4:** Causes of prosthesis failure

Type of complication	Type 1	Type 2	Type 3	Type 4	Type 5	All types
(n)	soft tissue	aseptic	structural	infection	tumor	
	failure	loosening	failure		progression	
Proximal femur (49)	0	1	2	1	5	
Distal femur (39)	0	8	1	4	6	
Proximal tibia (12)	0	1	0	1	1	
Overall (100)	0	10	3	6	12	31

No type 1 failure (soft tissue related) occurred. Type 2 failure (aseptic loosening) occurred in 10 patients (PF/DF/PT = 1/8/1). All cases were caused by loosening of the component due to rotational instability at an early phase (nine new cementless stems and one femoral component of PT). Six of the 10 patients underwent revision surgery, and four were treated conservatively. Type 3 failure (structural) occurred in three patients (PF/DF/PT = 2/1/0) and included hip dislocation (*n* = 1) and periprosthetic fracture (*n* = 2). All failures were treated surgically. No stem breakage was observed at the final follow-up. Type 4 failure (infections) occurred in six patients (PF/DF/PT = 1/4/1) and included early infection (*n* = 1) and late infection (*n* = 5). All failures were treated surgically and three of six patients underwent revision surgery. Type 5 failure (local tumor progression) occurred in 12 patients (PF/DF/PT = 5/6/1) and included skeletal recurrence (*n* = 5) and soft tissue recurrence (*n* = 7). Two patients (one with osteosarcoma and one undifferentiated pleomorphic sarcoma of bone) underwent amputations for local recurrences. Revision surgery was performed in 13 patients and amputation in two patients. Early implant loosening was significantly more common in the DF (*p* = 0.006) and straight stem of PF/DF (*p* = 0.038).

## Discussion

Ideally, reconstruction of bony defects should be biological. In large defects in curative limb salvage surgery, complete biological reconstruction is limited and, in most cases, large endoprostheses have been used. Long-term durability is required for the fixation of tumor endoprostheses to the diaphysis of long bones. However, the optimal method remains controversial. Previous reports have revealed that cemented prostheses have a higher incidence of aseptic loosening [4, 10, 13, 14], which has encouraged many orthopedic oncologists to use cementless stems [15, 16]. Early cementless stems incorporated side plate and screw components, which often resulted in various failures such as stem breakage, screw loosening, and stress shielding [15, 18, 19], as such, the development of a new cementless stem is required. KMLS has also developed a newly designed cementless stem with a unique tapered press-fit design for the PF, DF, and PT and has been in use since August 2014. From August 2014 to March 2018, 100 cases of KMLS newly designed cementless stems were prospectively registered for use at JMOG-affiliated institutions. We retrospectively reviewed short-term clinical outcomes of these cementless stems in March 2020 with a minimum follow-up of 2 years. The overall prosthesis survival rates at 2 and 4 years were 88.2 and 79.6%, respectively. The prosthesis-specific survival rate excluding infection and tumor recurrence at 2 and 4 years were 90.2 and 87.9%, respectively. The limb salvage rate at 2 and 4 years was 98.9 and 96.9%, respectively. The KMLS newly designed cementless stem showed good short-term results with a mean follow-up period of 35 months. Previous studies demonstrated that prosthetic survivals with cementless stem fixation ranged from 65.4 to 96% [13, 21, 22, 30, 31] but the heterogeneity of the patient population makes it difficult to directly compare the survival results of the present series with those of these previous studies. However, our results are comparable to those previously reported in the literature.

The design of this cementless stem has two features including the tapered press-fit design and the short fixation of the tapered portion. The previous cementless stems incorporated side plate and screw components. Various failures, such as stem breakage, screw loosening, and stress shielding, are often experienced and the tapered design to avoid stem breakage improved the strength of the stem base, where the most stress is concentrated, and there was no stem breakage in this cohort with a mean follow-up period of 35 months. However, Capanna *et al.* reported that six of 95 cases of femoral stem breakage occurred at an average of 43 months [17] thus, careful follow-up in the long term is needed. In addition, the tapered press-fit design allows early weight-bearing, which is advantageous for bone ingrowth, and makes the patient ambulatory at an early stage, which is a greatly beneficial to the patient.

**Fig. 5:**
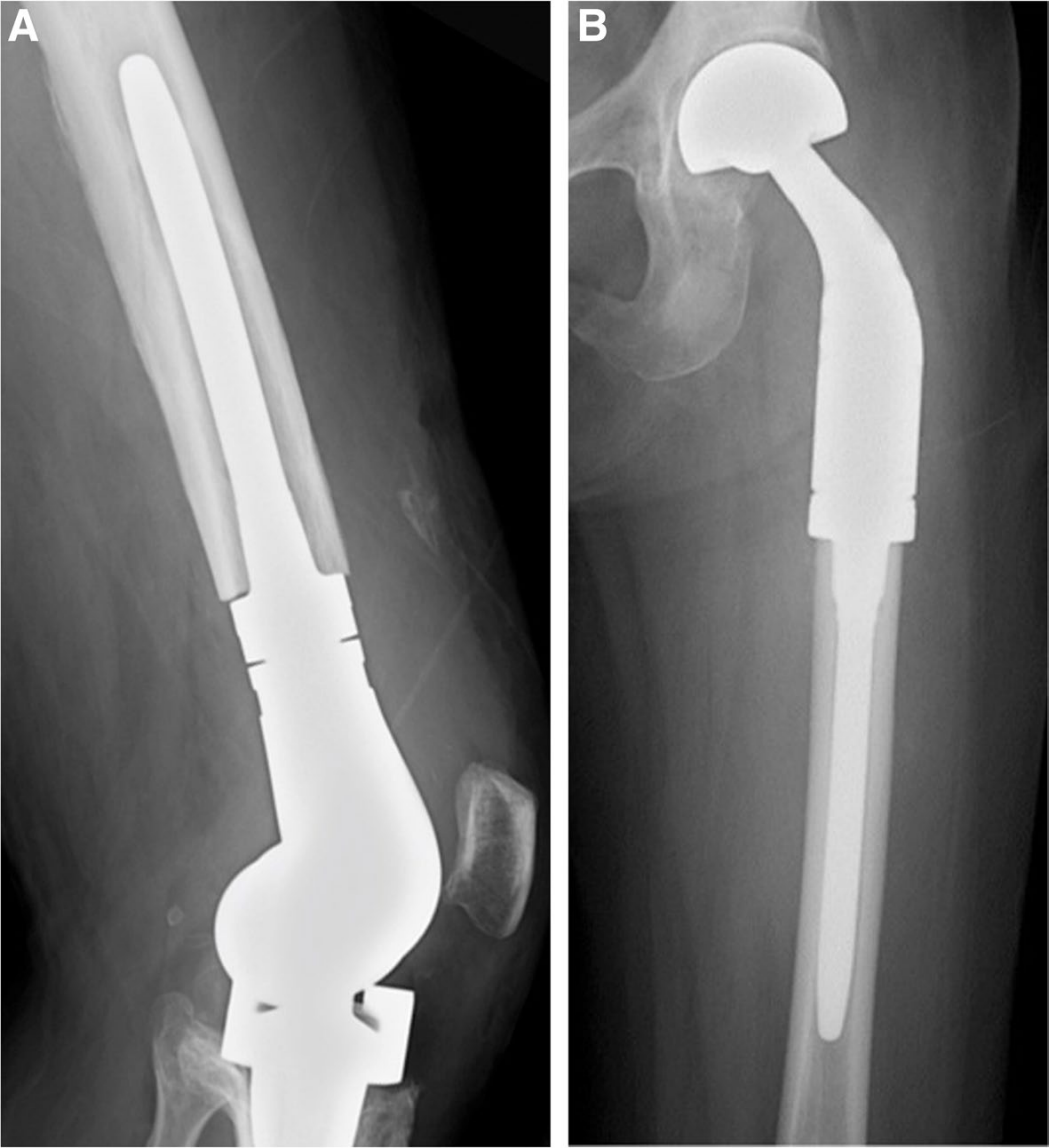
A 3-year postoperative radiograph showing the lateral view of the KMLS newly designed cementless stem in a patient with **A**. giant bone cell tumor and **B**. bone metastases from breast cancer. KMLS, Kyocera Modular Limb Salvage System

Cementless stems with long extensively porous-coated intramedullary stems provide strong anchorage over the whole length of the stem. However, intramedullary long fixation is not physiological and reduces the load on the surrounding cortical bone. This causes stress shielding of the surrounding bone, which leads to aseptic loosening. The short fixation design of this new cementless stem has been improved with a concept that preserves physiological bone loading and bone stock. Notably, the ISOLS radiographic evaluation at 2 years after surgery revealed very good bone remodeling and anchorage in most cases (bone remodeling: 90% / excellent and good, anchorage: 97% / excellent and good). Once initial rotational stability was achieved, there was little stress shielding, and the bone stock was preserved (Fig. 5A, B). In our experience, stress shielding often reaches a plateau at 2 years after surgery and stabilizes thereafter, but medium- to long-term follow-up is necessary.

Conversely, in total, 31 failures/complications (31%) were observed in 100 KMLS newly designed cementless stems. The prosthesis-specific survival rate excluding infection and tumor recurrence at 2 and 4 years were 90.2 and 87.9%, respectively. Younger age (*p* = 0.045) and primary tumor (*p* = 0.057) were associated with poor prognosis of prosthesis-specific survival excluding infection and tumor recurrence. Early loosening in nine cases was associated with the new cementless stem. This failure was significantly more common in the DF (*p* = 0.006) and PF/DF straight stem (*p* = 0.038). It is important to obtain the initial rotational stability in the anchorage of the cementless stem in this design. The DF is anatomically prone to rotational stress and is a common site for primary malignant bone tumors such as osteosarcoma, which is associated with younger age and high activity. Therefore, a short fixation with a tapered press-fit is not sufficient to provide reliable rotational stability for high-activity patients, and the use of a curved stem is recommended. Furthermore, the tapered press-fit for thin diaphysis of young patients is often difficult to broach the medullary canal without cracking. Cracking of the host bone might compromise bone healing, especially under additional intensive chemotherapy. Some failures with rotational instability occurred in the early stages of this study, but the reaming technique and the development of optional instruments allowing gradual deepening of the grooves in the cortical bone solved these problems over time. Although this is a problem with all cementless stems as a whole, this stem may be difficult to use in poor bone quality after long-term chemotherapy, because of the lack of initial fixation. In such cases, cemented stems that provide initial fixation or new cementless stem systems such as the Compress endoprosthesis [26] that can improve bone quality are options. Further investigations are required to confirm the superiority of the Compress endoprosthesis.

The length of the stem is fixed at 125 mm for the femoral component and 110 mm for the tibial component. Although proximal femoral reconstruction with arthroplasty shows acceptable primary stability as well as acceptable implant survival, aseptic loosening with less durability is reported after knee reconstruction within the same implant system [32]. Stem length and anatomical and biomechanical differences might influence implant survival. In this study, early implant loosening was significantly more common in the DF and PF/DF straight stem. However, conclusive biomechanical evidence for this issue is lacking. Nadorf et al. investigated an in vitro comparative study of two different tapered stems (100 and 160 mm) in reconstructive endoprosthesis after distal femoral resection and measured higher relative micromotions with a 160 mm longer stem and suggested the use of the shorter stem to be more favorable in case of primary implant fixation [33]. Because of the specific short fixation design of the stems in this study, further anatomic site-specific biomechanical studies are needed to determine the optimal stem length considering the current clinical results.

This study has some limitations. First, this was a retrospective review, and patient diagnoses are heterogeneous in terms of biological behavior and stage. Therefore, this study is subject to their inherent limitations and biases. However, KMLS newly designed cementless stems were prospectively registered at JMOG-affiliated institutions, and data on postoperative function and postoperative radiographic evaluation were collected. Endoprosthesis is a rare procedure and experience at a single institution is limited and it is difficult to perform a prospective evaluation with sufficient statistical power. We believe that it is significant to share the experience of newly designed endoprosthesis at an early stage in a multi-center setting for future development. Second, the duration of patient follow-up is short. In our series, the mean duration of patient follow-up was 35 months (range: 24–53 months). A careful follow-up in the long term will be necessary in the future. However, most failures at the bone-endoprosthesis junction of cementless stem that require revision surgery would be likely to occur very early in the postoperative period. Therefore, we set the registration period to 4 years and the minimum follow-up period to 2 years. Finally, anatomical locations (PF, DF, and PT) are strongly related to diagnosis (primary or metastasis) and age, and interpretation of prosthesis survival and complications should take these biases into account.

## Conclusion

The long-term fixation of tumor endoprosthesis to the diaphysis of the lower extremity remains challenging. JMOG has been involved in improving small and light modular prostheses that are suitable for Asian-pacific patients. KMLS have developed a newly designed cementless stem with a unique tapered press-fit and short fixation design for the PF, DF, and PT. This cementless stem showed good short-term results in preserving bone stock. The short fixation design of this cementless stem has been improved with a concept that preserves physiological bone loading and bone stock. The ISOLS radiographic evaluation at 2 years after surgery revealed very good bone remodeling and anchorage. On the other hand, we experienced several cases of early loosening. To prevent early loosening, straight stem should not be used in PF and DF and broaching in the medullary canal requires great care to avoid cracking the bone. In the future, careful long-term follow-up is necessary, and we should consider refining our cementless stem design to account for higher activity and anatomical locations where stress is concentrated, based on the results of this study.

## Data Availability

All data can be available at request by email to the corresponding author. The data that support the findings of this study are available on request from the corresponding author, [ST]. The data are not publicly available due to the restriction that it contains information that could compromise the privacy of research participants.

## Abbreviations

DF: Distal femur
JMOG: Japanese Musculoskeletal Oncology Group
KMLS: Kyocera Modular Limb Salvage System
PF: Proximal femur
PT: Proximal tibia
